# Medical pioneers in cyberspace: German practice owners advertising on the WWW

**DOI:** 10.2196/jmir.1.1.e2

**Published:** 1999-08-05

**Authors:** Claudia Schuh

**Keywords:** Established physicians, Internet, advertising, survey, marketing of health services, private sector, Germany, public relations, practice management

## Abstract

**Background:**

In the last few years, the number of Internet users has increased explosively. In the same way the number of Internet users has exploded, the costs in the public health sector have also increased. This resulted cost saving efforts by those responsible people in politics and medical administration. These economy measures have impacted in particular the established physicians. The current German practice owners are faced with an unknown economic situation and are forced to think and act like businessmen. Doubts arise concerning the age-old tradition of the advertising prohibition. Now advertisement is recognized as an important necessity.

**Objectives:**

This study was conducted to answer the following questions:

- Who are the pioneers among the German practice owners presenting themselves to the public with their own website?
- How do they differ from their colleagues not advertising on the WWW?
- What motives and expectations do they associate with their website?

**Methods:**

Built on a detailed analysis of the relevant German and international literature, hypotheses were developed which were empirically checked in the further course of the work. For this purpose, an online survey was conducted on the WWW among established German physicians with their own websites.

**Results:**

194 physicians participated and 159 valid questionnaires were included in the analysis. The study revealed the following results:

The age and sex distribution as well as the distribution of medical specialties in the examined group correspond to the expectations.

A high percentage of the respondents participated in a medical professional organization.

The median time in practice for practice age of the respondents was a little more than ten years.

Many of the websites have been online less than one year.

The following hypotheses could only partly be confirmed by the results of the survey:

Physicians from different specialties deal with their own website differently.

The Internet Familiarity of the physicians is responsible for the importance they attach to advertisement on the web, particularly to their own website.

Surprisingly, the attitude towards the advertising prohibition in Germany, apparently results less from economic considerations than from age-conditioned opinions.

The size of a medical practice did not influence the attitude of the physicians towards their own website. However, the type of practice in which a physician works played a crucial role in this context.

**Conclusion:**

At present, the importance of the Internet for recruiting new patients is still small, but we anticipate it will continue to expand in the future.

## Introduction

This article results from a diploma thesis in media sciences written at the Institut für Journalistik und Kommunikationsforschung, Hochschule für Musik und Theater Hannover, Germany.

In the last years, the number of Internet users has exploded. As the Internet becomes a greater factor in both the fields of media and of economy, new users get access, new research and consulting requirements and new markets arise, and all this in a breath-taking speed.

In the same way the number of Internet users has exploded, the costs in the public health sector have also increased during this time in Germany. This resulted in cost saving efforts by those responsible people in politics and administration. These economy measures have impacted in particular the established physicians. The German practice owners are faced with an unknown economic situation: competitive conditions resulting from empty cash-boxes and a competition for patients and budgets force the physicians to think and act like businessmen. Doubts arise concerning the age-old tradition of the advertising prohibition. Now advertising is recognized as an important necessity. In Germany, physicians advertising on the WWW has only recently become possible [1,2] and is still being discussed and debated by lawyers and judges as well as by the members of the medical organizations defending the age-old professional ethics prohibiting advertising [3,4,5].

Various reasons can be listed for these changes: First, there are juridical questions: the advertising prohibition breaks some rules of the German constitution [6,7,8,9,10,11,12] and the European convention on human rights [13]. Second, the increasing Europeanizing and globalization of economies and markets demands an adaptation of German rules to those of other countries in order to equalize competitive conditions among physicians [4]. Finally, the advertising prohibition obstructs competition between physicians in Germany, too, a fact which collides with the concept of a market economy [14,15].

This study also investigated the relationship of physicians to the information technology, their knowledge, use of computers and especially of the Internet. Prior research from other institutions showed that the use of computers and of the Internet among established physicians depends on the age of the physician. Compared to the "general" German Internet user (most of them are between 20 and 29 years old) [16,17], medical users are older with a median age of 45 years [18,19,20]. Another important consideration is the medical specialty of the physicians using the Internet: It has been shown that physicians from different specialties show a different affinity for the use of computers. This can be concluded from the number of practices delivering their quarterly cost overview electronically. Orthopedists, urologists, general practitioners, oto-laryngologists and gynecologists were highest, whereas radiologists, neurologists, psychotherapeutists, anaesthesists, and pathologists do not use computers for this purpose very often [21]. Male physicians use the Internet much more often than female doctors.

Additionally German physicians from large and partnership practices tend to be more frequent medical online users [19,22].

A number of hypotheses were been concluded from the theoretical background information:

1. Physicians with an "open-minded" attitude towards IT and Internet attach a higher importance to the own website than those who are less "open-minded."
2. Physicians who are familiar with IT and Internet attach a greater importance to their own website than those who are less familiar with IT and Internet do.
3. Younger physicians and physicians from younger practices are more open-minded towards a breakdown of the advertising prohibition than older physicians and physicians from older practices.
4. For physicians from younger practices, the motive of "winning new patients" is more important than for physicians from older practices.
5. Physicians from partnership practices and group practices attach a higher importance to advertising on the WWW than physicians from small and single practices.
6. Male physicians are much more likely than female physicians to have their own practice websites.
7. Physicians participate in professional medical politics and organizations are more "open-minded" towards a breakdown of the advertising prohibition than those who participate less.

## Materials and Methods

Between October 31^st^, 1998 and February 17^th^, 1999, a WWW survey was conducted. German, Austrian and Swiss established physicians were asked to participate by email, by several user net postings (in medical newsgroups and in a newsgroup announcing German web surveys) as well as with publications in German popular medical media and by hyperlinks from other medical websites. Dentists or physicians working in hospitals were **not** included in the survey.

The original questionnaire (in German language) can be visited at the http://unics.rrzn.uni-hannover.de/schuh/frabo01.html. It was developed using Netscape Communicator 4.06 and an evaluation version of Perseus Survey Solutions for the Web. The questionnaire was edited in simple HTML without any java applets or special scripts in order to enable a maximum of the addressed users to view it with their browsers.

The questionnaire consists of the following thematic groups of questions:

- the physician himself, his motives and attitudes
- the website, its age, size etc.
- feedback to the website
- the sociodemographical data of the respondents

In addition to the questionnaire itself, there was an introduction page saying hello to the user and introducing the study very briefly. (http://unics.rrzn.uni-hannover.de/schuh/index-alt.html) This introduction page indicated hyperlinks to a support page for those who had never filled out a WWW questionnaire before (http://unics.rrzn.uni-hannover.de/schuh/hilfe4.html) as well as to a "more information" page informing the interested reader about framework, goals and hypotheses of the study (http://unics.rrzn.uni-hannover.de/schuh/claudia.html). All pages contain a mail-to link that enabled the user to send an email to the author. After filling out the questionnaire and sending it to the author, a "thank you" page appears.

In order to guarantee anonymity to the respondents, the completed questionnaires were sent back to the author via a U.S. remailer provided by Perseus to all users of their software.

## Results

194 physicians participated, and 159 valid questionnaires were included in the analysis and furnished the following results, represented by the corresponding tables:

To a large extent, the age (Table 2) and the sex (Table 1) distribution in the examined group corresponded to the expectations from the general information on medical Internet users.

The distribution of the specialties in the examined group (Table 3) did not surprise us after the figures presented in the theoretical section of this paper.

The high percentage of physicians engaged in professional organizations (Table 14) was remarkable in the examined group.

More than 20 percent of the respondents held a position in a professional organization. The median time in practice of a little more than ten years (Table 6) with a substantially smaller modus of five years permits the assumption that the number of physicians who present their practice on the WWW will further grow in the future.

Due to the economic conditions for the operation of a medical practice, as expected especially young practices count on this new method of advertising. This can also be concluded from the fact that many of the websites of the respondents had only been online for less than one year (Table 12).

However, winning new patients is a less important motive for running a website than retaining existing patients by offering a new or better service to them.

**Table 1:** Sex Distribution

		frequency	%	valid %
**Valid**	Female	12	7.5	7.6
	Male	146	91.8	92.4
	Total	158	99.4	100.0
**Missing**		1	.6	
**Total**		159	100.0	

**Table 3:** Medical specialty

		Frequency	%	valid %
**Valid**	General Practitioner	54	34.0	34.4
	Anaesthesiology	2	1.3	1.3
	No Specialty	12	7.5	7.6
	Ophthalmology	5	3.1	3.2
	Surgery	3	1.9	1.9
	Dermatology	2	1.3	1.3
	Gynecology	13	8.2	8.3
	Otolaryngology	5	3.1	3.2
	Internal Medicine	23	14.5	14.6
	Pediatrics	3	1.9	1.9
	Head and Neck Surgery	3	1.9	1.9
	Neurology	5	3.1	3.2
	Orthopedics	9	5.7	5.7
	Psychiatry	2	1.3	1.3
	Radiology	2	1.3	1.3
	Urology	11	6.9	7.0
	Other	3	1.9	1.9
	Total	157	98.7	100.0
**Missing**		2	1.3	
**Total**		159	100.0	

**Table 2:** Age Distribution

		frequency	%	valid %
**Valid**	Less than 30 years	1	0.6	.6
	31-40 years	42	26.4	26.8
	41-50 years	82	51.6	52.2
	51 and + years	32	20.1	20.4
	Total	157	98.7	100.0
**Missing**		2	1.3	
**Total**		159	100.0	

**Table 6:** Time in Practice

		Frequency	%	valid %
**Valid**	Less than 5 years	32	20.1	20.3
	5 through less than 10 years	52	32.7	32.9
	10 through less than 15 years	33	20.8	20.9
	15 years or more	41	25.8	25.9
	Total	158	99.4	100.0
**Missing**		1	.6	
**Total**		159	100.0	

**Table 12:** Website Presence

		frequency	%
**Valid**	< 3 months	20	12.6
	3 through < 6 months	22	13.8
	6 months through < 1 year	41	25.8
	1 through < 1.5 years	35	22.0
	1.5 years or longer	41	25.8
	**Total**	159	100.0

**Table 14:** Participation in Medical Professional Politics

		**frequency**	**%**	**valid %**
**Valid**	No	65	40.9	42.2
	Yes, Attend meetings etc.	57	35.8	37.0
	Yes, Holding Office	32	20.1	20.8
	Total	154	96.9	100.0
**Missing**		5	3.1	
**Total**		**159**	**100.0**	

## Discussion

The hypotheses could only partly be confirmed by the results of the survey:

Physicians of different specialties deal with their own website differently. The specialty of a physician could be considered an indicator to how and how intensively he uses the advertising method of a website. It can be assumed that apart from the IT- and Internet open-mindedness, there are other decisive factors that were not examined in this study.

The Internet familiarity of the physicians (Table 10) is responsible for the importance they attach to advertisement on the web and particularly to the own website.

This hypothesis could be confirmed and suggests at the same time that the WWW will gain in importance as an advertising medium for established physicians. More than a third of the respondents had used the Internet for less than two years (Table 9); with the explosive growth of the Internet the number of new physician users will increase and with the growth, develop new websites.

Surprisingly, the attitude towards the advertising prohibition results apparently less from economic considerations than from age-conditioned opinions. The key factor is the age of the physician, not the age of the practice, although naturally young physicians tend to be associated with younger practices. Not surprisingly, the tendency to try winning new patients by means of advertisement and a pronounced economic consciousness are stronger among owners of younger practices.

The size of a medical practice (Table 5) did not influence the attitude of the physicians towards their own website. However, the type of practice in which a physician works played a crucial role in this context (Table 4).

**Table 10:** Internet Familiarity (Scale)

		frequency	%	valid %
**Valid**	2: Not familiar	3	1.9	1.9
	3	5	3.1	3.2
	4	21	13.2	13.3
	5	35	22.0	22.2
	6	52	32.7	32.9
	7	19	11.9	12.0
	8	11	6.9	7.0
	9: Very familiar	12	7.5	7.6
	Total	158	99.4	100.0
**Missing**		1	.6	
**Total**		159	100.0	

**Table 9:** Use of Internet

		frequency	%	valid %
**Valid**	Less than 6 months	6	3.8	3.8
	6 months through less than 1 year	14	8.8	8.9
	1 through less than 2 years	35	22.0	22.2
	More than 2 years	103	64.8	65.2
	Total	158	99.4	100.0
**Missing**		1	.6	
**Total**		159	100.0	

**Table 5:** Size of Practice

		frequency	%	valid %
**Valid**	One of the 5 largest	58	36.5	44.6
	Medium	57	35.8	43.8
	Small	15	9.4	11.5
	Total	130	81.8	100.0
**Missing**		29	18.2	
**Total**		159	100.0	

**Table 4:** Type of Practice

		frequency	%	valid %
**Valid**	Single practice	98	61.6	62.0
	Partnership practice	59	37.1	37.3
	Partnership with other medical professions (Group)	1	.6	.6
	Total	158	99.4	100.0
**Missing**		1	.6	
**Total**		159	100.0	

### Conclusion

In conclusion, it appears that the importance of the Internet for the recruitment of new patients is still small, but it will continue to expand in the future. This is indicated the results presented in this study, by the general development of the Internet, by the European direction of markets and juridical systems, and by the examples from other countries. The few website operators among the established physicians are doing the pioneering work in Cyberspace. It is hoped for them - the respondents of this survey - that their pioneer spirit will be rewarded.

## Abbreviations

HTML: Hypertext Markup Language
IT: Information Technology
WWW: World Wide Web
